# Kidney transplant-related knowledge and influencing factors in Chinese kidney transplant candidates and recipients: A cross-sectional study

**DOI:** 10.3389/fpubh.2023.1027715

**Published:** 2023-03-03

**Authors:** Hangxia Ma, Maosen Hu, Jingjing Wan

**Affiliations:** ^1^Xiangya Nursing School, Central South University, Changsha, Hunan, China; ^2^The Third Xiangya Hospital, Central South University, Changsha, Hunan, China; ^3^Nursing Department, Outpatient and Emergency Operating Room, The Third Xiangya Hospital, Central South University, Changsha, Hunan, China

**Keywords:** kidney transplantation, knowledge, health education, candidates, recipients

## Abstract

**Objective:**

To investigate the kidney transplantation knowledge of kidney transplant (KT) candidates and recipients, and to explore the related influencing factors.

**Methods:**

From March to July 2022, a total of 170 KT candidates and 270 KT recipients were investigated in two tertiary and Grade A hospitals in Hunan Province, China, using demographic questionnaires and the Kidney Transplant Understanding Tool (K-TUT). Multiple linear regression was used to explore the influencing factors of related knowledge of kidney transplantation.

**Results:**

The scores of kidney transplantation knowledge of the two groups were 50.67 (Ranged: 0–63) and 52.79 (Ranged: 0–62), indicating a middle level of knowledge. Education level and whether they have received health education were significantly associated with the knowledge level of kidney transplantation in both KT candidates and recipients. In addition, age and fertility status were only significantly associated with the knowledge level of kidney transplantation in KT recipients.

**Conclusion:**

Our finding shows that the knowledge level of KT candidates and recipients is not optimistic, which suggests that healthcare providers should pay more attention to the health education of this population. In addition, future health education interventions should consider the education level, age, and fertility status factors affecting kidney transplantation knowledge in KT candidates and recipients.

## 1. Introduction

Chronic kidney disease (CKD) refers to the structural and functional abnormalities of kidney caused by various reasons (1). At present, it has become a global public health problem. Epidemiological surveys show that the global prevalence can reach 10–15%, and the incidence is increasing year by year (2, 3). The prevalence of CKD in China was reported to be 10.8%, of which 1–3% would become End-Stage Renal Disease (ESRD) (4). Globally, the number of patients with ESRD is increasing by 7% per year (5). ESRD is a progressive disease that requires prompt renal replacement therapy to prevent death. The disease not only leads to increased hospitalization rates and health care costs, but also a 20–50% mortality rate within 24 months (6). Kidney transplantation is the best treatment for ESRD. According to the World Health Organization's Global Organ Donation and Transplantation data, a total of 77,319 kidney transplants were performed worldwide by 2017, of which 10,793, or 13.95 percent, were performed in China, ranking second in the world (7). As one of renal replacement therapy, kidney transplantation can effectively improve the survival rate and quality of life of patients with CKD, and significantly reduce the cost of medical care, which is also recognized as the first choice of treatment (8, 9). But post-transplant patients need to have sufficient knowledge of immunosuppressive drugs, health management, infection prevention, and transplant rejection symptoms to take care of themselves (10). Studies had shown that the lack of transplant-related knowledge in KT candidates could cause preoperative anxiety, fear and lack of mental preparation for the postoperative situation (11, 12). For KT recipients, lack of transplant related knowledge would lead to postoperative infection, and even death in severe cases (13).

Therefore, it is very important to understand the transplant related knowledge level of KT candidates and recipients, so as to carry out targeted health education. At present, in China, there is still a lack of research on the transplantation knowledge of KT candidates, or the self-made scale is used to study the transplantation knowledge level of KT recipients, which is not scientific and reliable (14, 15). This study investigated the current status of transplant knowledge of KT candidates and recipients in China by using the Kidney Transplant Understanding Tool of which the Chinese validity and reliability study was conducted by the same authors (16), and to find out its weak points and influencing factors of their kidney transplantation knowledge, so as to provide a basis for formulating targeted health education.

## 2. Methods

### 2.1. Participants

Participants were recruited by convenience sampling from March to July 2022 from two tertiary and Grade A hospitals in Hunan Province, China. They met the following inclusion criteria: (a): KT recipients and candidates; and (b) ability to read and communicate effectively; and (c) informed consent and voluntary participation in the study. Patients were excluded from the study if they were with mental illness or cognitive impairment and with ≥ 2 or more kidney transplants. According to Kendall's sample size calculation principle, the sample size of the reliability and validity test is 5–10 times of the number of evaluation tool items (17), and the minimum sample size required for this study is 110 participants each. At the same time, according to the requirement that the sample size should be 10–20 times of the independent variable, and considering the sample loss rate of 10%, the minimum sample size required for this study was 132 participants each (18). Finally, a total of 170 KT candidates and 270 KT recipients were included in this study, a total of 440 participants.

### 2.2. Ethical consideration

The study was approved by the Ethics Committee of Xiangya School of Nursing, Central South University on March 15, 2022 (E202230), with permission and approval from the hospital management, and conformed to the Helsinki Declaration of Ethical Principles for Medical Research. Informed consent was obtained from all participants, participation was voluntary, there were no incentives, they had the right to withdraw from the study at any time, and they were assured that the data would only be used by the research team.

### 2.3. Questionnaire

The questionnaire included two parts: the first part was the general information of the participants, including age, gender, education level, religious belief, place of residence, occupation, family per capita monthly income, marital status, waiting time for kidney transplantation, and time after transplantation. The second part was the K-TUT, by the Canadian scholar Mansell developed in 2017, mainly to determine the knowledge of KT recipients and candidates in a healthy lifestyle and compliance with medical plans, including concepts related to KT, taking immunosuppressants, identification and prevention of complications, physical changes after KT, traditional treatment measures and infections, pregnancy and sexual health, etc. The tool has 22 questions (9 judgment questions and 13 multiple topics), and multiple choice questions have more than one correct answers. Each option can be considered as a judgment question, and the tool will be transformed into 69 judgment questions. The rule is 1 point for correct answer and 0 point for wrong answer, and the total score is 69 points. The higher the score, the better the patient's understanding of KT related knowledge. It is currently applied in the Korea (10), United States (19), North America (20), Iran (21), Canada (22), etc. The Cronbach's alpha of the tool is 0.79 to 0.88, and the intra-group correlation coefficient is 0.76 to 0.94, which has a good reliability (23). Our research team carried out the Chinese version of the tool. The S-CVI of the Chinese version of KTUT was 0.967, and the I-CVI of each item ranged from 0.8 to 1.0. The internal consistency reliability and retest reliability of the Chinese version of K-TUT were 0.778 and 0.902, respectively, for KT candidates. For KT recipients, the internal consistency reliability and retest reliability of the Chinese version of K-TUT were 0.769 and 0.888, respectively (16).

### 2.4. Data analysis

SPSS 26.0 was used for statistical analysis. Demographic were described by frequencies, and scores were described by the mean (M) ± standard deviation (SD). The dependent variables all conform to normal distribution. The main methods included descriptive analysis, sample *t-*test, analysis of variance and multiple regression analysis. The significant level of all indexes was set at α = 0.05.

## 3. Results

### 3.1. Socio-demographic characteristics

Among the 440 participants in our study, there were 170 KT candidates and 270 KT recipients. For KT candidates, male (57.1%) was slightly higher than female (42.9%). What's more, most of the patients were Han nationality (94.7%) and had no religious belief (95.9%). About one-third of the patients were from rural areas (30%) and the rest were from cities; see the Table 1 for other basic information. For recipients, the proportions of gender, nation, religious belief and residence were similar to those of the recipients. See the Table 2 for other basic information.

**Table 1:** Demographic characteristics of KT candidates.

**Items**	**KT candidates *n* (%)**	**Scores**	**t/F**	***P***
**Gender**			−1.300	0.196
Male	97 (57.1)	50.11 ± 6.174		
Female	73 (42.9)	51.30 ± 5.512		
**Age**			2.188	**0.030**
≤ 45	93 (54.7)	51.52 ± 5.774		
>45	77 (45.3)	49.55 ± 5.933		
**Nation**			−0.601	0.549
Han	161 (94.7)	50.56 ± 5.991		
Ethnic minority	9 (5.3)	51.78 ± 4.324		
**Religion**			1.665	0.098
No	163 (95.9)	50.78 ± 5.792		
Yes	7 (4.1)	47.00 ± 7.916		
**Place of residence**			−2.287	**0.023**
Rural	51 (30)	49.06 ± 6.345		
City	119 (70)	51.29 ± 5.611		
**Education level**			6.037	**< 0.001**
Primary school	4 (2.4)	47.25 ± 5.679		
Junior high school	47 (27.6)	48.28 ± 5.136		
High school/secondary Specialized school	44 (25.9)	49.70 ± 6.129		
Undergraduate/ junior college	68 (40)	52.54 ± 5.538		
Postgraduate	7 (4.1)	55.43 ± 5.350		
**Occupation**			3.424	**0.001**
Medical staff	3 (1.8)	60.67 ± 7.572		
Business staff	33 (19.4)	53.30 ± 5.312		
Civil servants	5 (2.9)	47.60 ± 8.620		
Teacher	9 (5.3)	51.11 ± 5.645		
Self-employed	39 (22.9)	49.85 ± 6.081		
Student	4 (2.4)	53.25 ± 4.349		
Workers	14 (8.2)	48.93 ± 5.076		
Farmer	23 (13.5)	47.83 ± 5.237		
None	40 (23.5)	50.63 ± 5.256		
**Marital status**			1.956	0.145
Single	30 (17.6)	52.03 ± 6.718		
Married	135 (79.4)	50.19 ± 5.728		
Divorced	5 (2.9)	53.80 ± 4.025		
Widowhood	0			
**Fertility status**			1.138	0.343
None	40 (23.5)	51.93 ± 6.120		
One girl	33 (19.4)	50.27 ± 4.824		
One boy	47 (27.6)	49.26 ± 5.940		
One boy and girl	26 (15.3)	50.23 ± 7.607		
Two girls	9 (5.3)	52.33 ± 4.123		
Two boys	12 (7.1)	52.50 ± 4.739		
Others	3 (1.8)	49.33 ± 2.309		
**Monthly household income per capita**			2.644	**0.035**
1,000 以下	17 (10)	47.24 ± 6.160		
1,000–3,000	31 (18.2)	49.65 ± 5.024		
3,000–5,000	38 (22.4)	50.61 ± 7.262		
5,000–8,000	31 (18.2)	50.81 ± 5.388		
8,000 以上	53 (31.2)	52.19 ± 5.122		
**Renal waiting time**			0.421	0.794
< 1 year	102 (60)	50.32 ± 6.121		
1–3 years	43 (25.3)	51.33 ± 5.723		
3–5 years	10 (5.9)	50.20 ± 4.517		
5–10 years	11 (6.5)	51.73 ± 4.982		
More than 10 years	4 (2.4)	48.75 ± 9.215		
**Health education**			4.447	**< 0.001**
Yes	120 (70.6)	51.86 ± 5.325		
No	50 (29.4)	47.66 ± 6.242		
Knowledge scores	50.67 ± 5.912			

The bold values indicated P **<** 0.05.

**Table 2:** Demographic characteristics of KT recipients.

**Items**	**KT recipients *n* (%)**	**Scores**	**t/F**	***P***
**Gender**			−1.677	0.095
Male	152 (56.3)	50.11 ± 6.174		
Female	118 (43.7)	51.30 ± 5.512		
**Age**			4.647	**< 0.001**
≤ 45	168 (62.2)	53.99 ± 5.265		
>45	102 (37.8)	50.81 ± 5.721		
**Nation**			−0.662	0.508
Han	240 (88.9)	52.71 ± 5.666		
Ethnic minority	30 (11.1)	53.43 ± 5.538		
**Religion**			0.964	0.336
No	260 (96.3)	52.85 ± 5.572		
Yes	10 (3.7)	51.10 ± 7.475		
**Place of residence**			−2.227	**0.027**
Rural	95 (35.2)	51.76 ± 5.163		
City	175 (64.8)	53.35 ± 5.830		
**Education level**			5.924	**< 0.001**
Primary school	7 (2.6)	49.71 ± 4.152		
Junior high school	55 (20.4)	50.96 ± 5.246		
High school/secondary Specialized school	83 (30.7)	51.71± 5.009		
Undergraduate/junior college	112 (41.5)	54.50 ± 5.606		
Postgraduate	13 (4.8)	54.31 ± 7.836		
**Occupation**			2.770	**0.006**
Medical staff	9 (3.3)	58.11 ± 7.079		
Business staff	46 (17.0)	53.39 ± 5.607		
Civil servants	18 (6.7)	52.72 ± 6.884		
Teacher	24 (8.9)	53.13 ± 5.343		
Self-employed	46 (17.0)	52.80 ± 5.924		
Student	7 (2.6)	54.43 ± 3.155		
Workers	20 (7.4)	50.70 ± 4.624		
Farmer	29 (10.7)	49.72 ± 4.284		
None	71 (26.3)	53.30 ± 5.434		
**Marital status**			0.939	0.422
Single	53 (19.6)	53.77 ± 5.067		
Married	196 (72.6)	52.65 ± 5.701		
Divorced	19 (7.0)	51.79 ± 6.588		
Widowhood	2 (0.7)	50.00 ± 5.657		
**Fertility status**			3.406	**0.003**
None	75 (27.8)	54.24 ± 5.151		
One girl	57 (21.1)	53.07 ± 4.982		
One boy	68 (25.2)	52.28 ± 5.550		
One boy and girl	36 (13.3)	53.58 ± 5.400		
Two girls	17 (6.3)	49.35 ± 7.541		
Two boys	9 (3.3)	49.22 ± 6.515		
Others	8 (3.0)	49.25 ± 6.205		
**Monthly household income per capita**			3.375	**0.010**
1,000 以下	18 (6.7)	52.06 ± 5.765		
1,000–3,000	64 (23.7)	51.55 ± 5.489		
3,000–5,000	70 (25.9)	51.97 ± 5.907		
5,000–8,000	58 (21.5)	53.28 ± 4.902		
8,000 以上	60 (22.2)	54.82 ± 5.703		
**Renal waiting time**			1.365	0.254
< 1 year	169 (62.6)	52.35 ± 5.284		
1–3 years	86 (31.9)	53.73 ± 6.307		
3–5 years	10 (3.7)	53.10 ± 6.244		
e 5–10 years	5 (1.9)	50.80 ± 2.387		
More than 10 years	0	0		
**Time after kidney transplantation**			1.876	0.099
< 3 months	27 (10.0)	55.26 ± 5.502		
3 months−1 year	44 (16.3)	51.43 ± 4.795		
1–3 years	61 (22.6)	52.48 ± 6.305		
3–5 years	39 (14.4)	52.36 ± 5.183		
5–10 years	61 (22.6)	53.49 ± 5.781		
More than 10 years	38 (14.1)	52.42 ± 5.436		
**Health education**			4.447	**< 0.001**
Yes	248 (91.9)	51.86 ± 5.325		
No	22 (8.1)	47.66 ± 6.242		
Knowledge scores	52.79 + −5.646			

The bold values indicated P **<** 0.05.

### 3.2. Univariate analysis of influencing KT related knowledge

The results showed that there were significant differences in age, place of residence, education level, occupation, family per capita monthly income, and whether the patients received health education among KT candidates (*P* < 0.05). To be specific, patients with age ≤ 45, living in cities, higher education level, medical staff, higher family per capita monthly income, and receiving health education had higher knowledge level of kidney transplantation, and their knowledge mean score was 50.67 (63), as detailed in the Table 1. There were significant differences in age, place of residence, education level, occupation, birth status, family per capita monthly income, and whether to receive health education among KT recipients (*P* < 0.05), and the specific results are similar to those of KT candidates. Their knowledge mean score was 52.79 (62), as detailed in the Table 2.

### 3.3. Multivariate analysis of influencing KT related knowledge

The K-TUT scores of KT candidates and recipients were considered as dependent variables. Statistically significant data in general data (age, place of residence, education level, occupation, fertility status, family per capita monthly income, and health education) were considered as independent variables. Multiple linear regression models were established respectively. The results showed that the two independent variables of education level and whether to receive health education affected the transplant-related knowledge level of KT candidates (see Table 3). The details were as follows: Education level and whether they have received health education have a positive association on the knowledge level of kidney transplantation. The higher the education level, the higher the knowledge level of KT candidates, and the level of kidney transplantation was higher in KT candidates who had received health education (B = 1.589, SE = 0.507, *p*-value = 0.002; B = −4.663, SE = 0.889, *p*-value = 0.000). The results showed that the four independent variables, age, education level, fertility status and whether to receive health education, affect the transplant-related knowledge level of KT recipients (see Table 4). Specifically, the influence of education level and whether they have received health education on the knowledge level of KT recipients is similar to that of KT candidates (B = 0.867, SE = 0.415, *p*-value = 0.038; B = −5.884, SE = 1.126, *p*-value = 0.000). The difference is that age and fertility status have a negative association on the knowledge level of KT recipients. The older they were, the more children they had, and the lower the knowledge level of KT recipients.

**Table 3:** Multiple linear regression of KT candidates.

**Items**	**B**	**SE**	**β**	**t**	***P***
Constant	51.912	3.427	–	15.148	**< 0.001**
Age	−1.409	0.878	−0.119	−1.605	0.111
Place of residence	0.713	0.948	0.055	0.753	0.453
Education level	1.589	0.507	0.257	3.133	**0.002**
Occupation	−0.126	0.169	−0.056	−0.741	0.459
Monthly household income per capita	0.376	0.323	0.086	1.163	0.247
Health education	−4.663	0.889	−0.360	−5.248	**< 0.001**

R^2^, 0.269; Adjusted R^2^, 0.242; F, 10.013; P < 0.001.

The bold values indicated P **<** 0.05.

**Table 4:** Multiple linear regression of KT recipients.

**Items**	**B**	**SE**	**β**	***t***	***P***
Constant	57.607	2.691	–	21.404	**< 0.001**
Age	−2.115	0.690	−0.182	−3.066	**0.002**
Place of residence	0.781	0.706	0.066	1.106	0.270
Education level	0.867	0.415	0.142	2.091	**0.038**
Occupation	0.001	0.134	0.001	0.008	0.994
Fertility status	−0.439	0.210	−0.121	−2.092	**0.037**
Monthly household income per capita	0.467	0.282	0.102	1.656	0.099
Health education	−5.884	1.126	−0.286	−5.224	**< 0.001**

R^2^, 0.240; Adjusted R^2^, 0.220; F, 11.849; P < 0.001.

The bold values indicated P **<** 0.05.

## 4. Discussion

This study investigated the knowledge level and associated factors of kidney transplantation among Chinese KT candidates and recipients through K-TUT after Chinese translation. The results showed that the average K-TUT score of KT candidates was 50.67 (63 points), while the average K-TUT score of KT recipients was slightly higher than that of KT candidates, which was 52.79 (62 points). This is consistent with the results of Rosaasen and Kang et al. (10, 23). Both in the medium level, but the accuracy is < 10% of individual items, such as entry “Every person who receives a kidney transplant feels better than they did before the transplant,” “Your creatinine will always be normal after your kidney transplant,” etc., showed that most patients see only positive aspects of KT, and ignore the negative aspects of transplantation or the importance of self-care, highlights the importance of health education. Therefore, in the future, medical staff should carry out targeted health education to further improve their knowledge level.

It was worth noting that this study showed that there are similarities and differences in factors affecting the knowledge level of KT recipients and candidates. Education level and health education were correlated with the knowledge level of kidney transplantation. Education level had a significant positive predictive effect on the knowledge level of renal transplantation. Several studies have arrived at similar conclusions (19, 24). This may be because patients with high education level are good at using various channels and opportunities to acquire knowledge, and have stronger ability to understand, accept and process knowledge and information. Good learning ability and strong knowledge seeking ability will have a positive impact on their knowledge level. However, patients with low education level have less ways and means to receive knowledge and information, and have no strong desire to understand the knowledge related to kidney transplantation, so they have weak cognition of the knowledge related to kidney transplantation (25, 26). This suggests that medical personnel should formulate personalized plans for people with different education levels when conducting health education. For example, for patients with low education levels, they can conduct propaganda and education through pictures, videos and other easy to understand ways, and regularly give feedback or carry out patient exchanges (27). Patients with high knowledge scores can share their own life habits, spread knowledge, and obtain positive stimulation among patients, so as to improve the knowledge level of kidney transplantation (13, 28). This study shows that patients who have received health education have a higher level of knowledge about kidney transplantation, which is consistent with the study. This may be because health education is an important way to acquire knowledge. After health education, patients can understand and master the knowledge of kidney transplantation more easily (29), which suggests that it is very important for medical staff to implement health education for patients.

The study also confirmed that age and fertility status had significant effects on KT recipients (*P* < 0.05), while these two factors had no effect on KT candidates. Specifically, the level of KT knowledge of KT recipients aged >45 years is higher than that of those aged < 45 years, this may be because with the increase of age, the memory and cognitive ability of the patients decrease, the ability to accept the knowledge related to renal transplantation is poor, and the access to medical care knowledge is also less; On the other hand, with the change of health concepts and the development of network information, young people pay more and more attention to their own health, are more willing to take the initiative to acquire health-related knowledge through the Internet and other ways, and have stronger ability to accept new knowledge and new ideas (30). This suggests that medical staff should try their best to use concise and clear language when carrying out health education for older patients. Key knowledge can be explained repeatedly to facilitate patients to strengthen their memory. Health education for young patients can be integrated with new media means to meet their needs. In addition, fertility status has a negative impact on the knowledge level of KT recipients. This is consistent with the study of Sun et al. (31). This may be because the increase in the number of children has put great financial pressure on families and strained resources. In the long run, this limits patients' access to important health promotion resources. This suggests that medical staff should pay more attention to KT recipients with a large number of children in the future.

This study has some limitations. First, the sample size of this study was small, which may affect the validity of the study results. Subsequent large cross-sectional studies should be conducted to explore the effectiveness of the Chinese-translated tools in this study. In addition, convenience sampling was used to select participants in this study, which may have a certain impact on the representativeness of the sample. In the future, the results of the survey and analysis should be based on large samples. The advantage of this study is that it is the first time in China to verify the K-TUT in the KT candidates and recipients. It provides a reliable tool for China to investigate the related knowledge level, analyze its influencing factors, and provide reference for formulating targeted health education and improving its knowledge level.

## 5. Conclusion

In conclusion, our results show that the Chinese version of the K-TUT has good reliability and validity, and the knowledge of KT candidates and recipients in China is at a medium level. Education level and whether they have received health education are the influencing factors of KT knowledge. In addition, age and fertility status will affect the KT knowledge level of KT recipients. This article can help medical staff to screen the weak points of kidney transplantation knowledge of patients, provide reference for the development of targeted health education, and suggest that medical staff can also start from the above factors to improve their knowledge level of kidney transplantation.

## Data availability statement

The original contributions presented in the study are included in the article/supplementary material, further inquiries can be directed to the corresponding author.

## Ethics statement

The studies involving human participants were reviewed and approved by Xiangya School of Nursing, Central South University. The patients/participants provided their written informed consent to participate in this study.

## Author contributions

HM and JW: conception, design of study, and drafting the manuscript. HM and MH: data collection and data analysis. All authors contributed to the article and approved the submitted version.
